# Histological Regression of Giant Cell Tumor of Bone Following RANK Ligand Inhibition

**DOI:** 10.1177/2324709614560216

**Published:** 2014-11-23

**Authors:** Martin F. Dietrich, Dominick Cavuoti, Michael Landay, Yull E. Arriaga

**Affiliations:** 1 University of Texas Southwestern Medical Center, Dallas, TX, USA

**Keywords:** Giant cell tumor, RANK ligand, denosumab, lung metastases

## Abstract

Lung metastases are a rare complication of giant cell tumors of bone. We herein describe an interesting case of histological regression and size reduction of lung metastases originating from a primary giant cell tumor of bone in response to the RANK ligand inhibitor denosumab.

## Case Description

A 37-year-old woman with a past medical history of type 2 diabetes mellitus and a complete resection of a giant cell tumor of the right femoral diaphysis presented to our clinic for initial evaluation after enlarging pulmonary nodules were discovered on surveillance imaging. Two years prior to referral to our clinic, she had undergone resection of an incidentally discovered giant cell tumor of the right distal femur. The maximum dimension of the tumor was 5 cm, and a 1.2-cm negative surgical resection margin was reported. The extent of resection and invasion of articular space required total replacement of the knee. Computed tomography (CT) of the chest at the time of the initial diagnosis revealed several subcentimeter nodules that were not amenable to biopsy by CT guidance or endobronchial ultrasound. The patient endorsed chronic cough but denied sputum production, hemoptysis, fever, or other symptoms. The patient’s family history was negative for malignancy; in particular, no bone tumors were reported. Her physical exam revealed changes consistent with total joint replacement of the right knee but was otherwise unremarkable. Her blood laboratory testing was unrevealing. Bone scan at diagnosis revealed persistent local radiotracer uptake at the site of the right knee, likely related to surgical intervention. Local radiotracer uptake resolved on subsequent bone scans. The patient had periodic cross-sectional imaging studies of the chest for assessment of the initially discovered lung nodules. After a period of 1.5 years of no growth, several lung nodules had significantly enlarged on chest imaging. Based on CT of the chest, abdomen, and pelvis as well as a nuclear bone scan, the lungs were the only site of metastatic involvement. To confirm the suspicion of lung metastatic disease, the patient underwent CT-guided transthoracic core biopsy of one of the lung nodules. Histology showed metastatic giant cell tumor with morphological features similar to the patient’s right femur mass.

**Figure 1.**
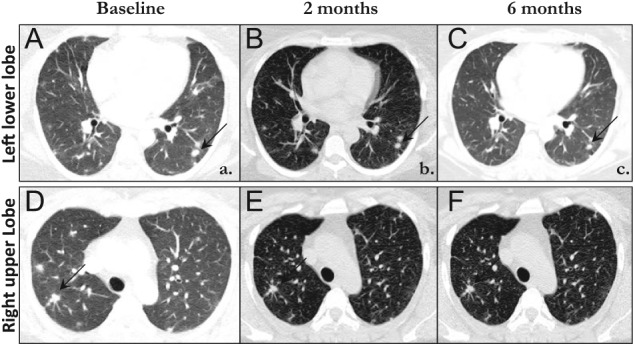
Computed tomography images of the chest are shown at baseline, 2, and 6 months after initiation of treatment with denosumab. Two representative lesions in the right upper lobe and left lower lobe are shown. The size of the shown lung nodules are smaller at 2 months of (B and E) treatment compared with baseline (A and D). No further shrinkage is seen after 4 additional months of treatment (C and F).

**Figure 2.**
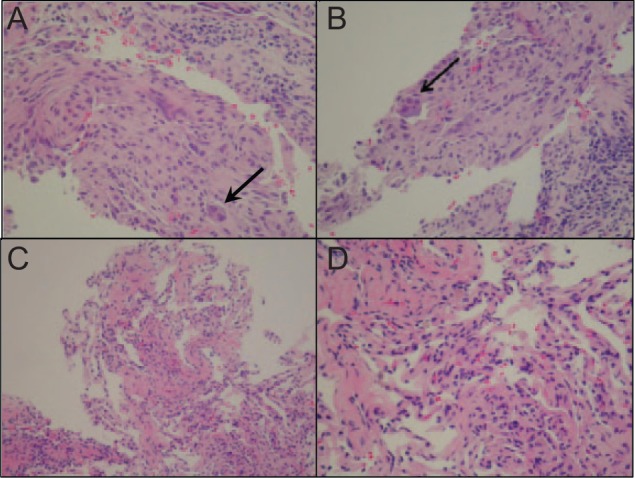
Hematoxylin and eosin stains of lung nodules in a patient with known giant cell tumor of the right femur. Shown here are representative sections obtained by core biopsy under computed tomography guidance. (A) and (B) demonstrate the classic giant cell formations prior to initiation of therapy with denosumab. Images (C) and (D) demonstrate unremarkable lung tissue after 2 months of weekly denosumab, with giant cells no longer appreciated histologically. Magnifications shown are 100-fold in (A) and (C) and 200-fold in (B) and (D), respectively.

The patient’s case was presented in our institutional tumor board, and a consensus decision was reached, recommending therapy with denosumab, based on results from phase II trials demonstrating safety and efficacy of denosumab in the treatment of metastatic giant cell tumors of bone (GCTBs).^1,2^ After comprehensive dental treatment, the patient was prescribed denosumab 120 mg via subcutaneous injection, with weekly loading doses on days 1, 8, and 15 of a 28-day cycle and then switched to 1 injection every 28 days.^2^ After 2 cycles of denosumab, contrast-enhanced CT scan of the chest showed reduction in size and number of all previously enlarged nodules, consistent with a partial response by RECIST 2.0 criteria (Figure 1). Several of these lung nodules that were not calcified initially became calcified on radiographic follow-up. A CT-guided core biopsy of one of the nodules after 2 cycles of denosumab showed histological response, with absence of giant cells (Figure 2). The patient received additional injections of denosumab without further shrinkage of the lung nodules after 4 and 6 cycles, respectively. She reported a significant improvement in her cough. There were no side effects noted or reported. The patient did not have denosumab-related serious adverse events such as osteonecrosis of the jaw or hypocalcemia. Currently, she receives maintenance subcutaneous injections of denosumab 120 mg every 28 days. Additionally, she has a physical examination and evaluation for denosumab-related toxicity every 3 months. Contrast-enhanced cross-sectional imaging of the chest is done every 6 months.

## Discussion

GCTBs constitute only 5% of primary bone tumors.^3^ Metastatic involvement of the lung is a rare complication of GCTBs.^4^ Although generally considered benign, patients with giant cell tumors can have locally invasive tumors with significant osteolytic potential. Cases of malignant transformation of GCTBs, specifically sarcomatous transformation, have been reported.^5,6^ The average rate of malignant transformation is very low; higher incidences of malignant transformation have been reported with previous irradiation of the involved site where the primary giant tumor arose.^7^ A clear understanding of the steps leading to spread and malignant transformation of GCTBs is lacking. Secondary genetic events, including changes in p53 status^3,5,8^ and amplification of c-myc,^9^ have been implicated. C-myc status further seems to correlate with development of pulmonary metastases.^9^ To our knowledge, no histological transformation of lung metastatic lesions has been reported in the literature.

For patients with local bone disease, wide local excision with orthopedic reconstruction is the current standard of care.^10^ For patients with metastatic or unresectable disease, a variety of systemic agents, including bisphosphonates,^11^ cisplatin/doxorubicin,^12^ and interferon α 2a,^13^ have been utilized and reported in the literature, with varying efficacy. Because of the low incidence of GCTBs and high cure rates with local therapy, there are no phase III clinical trials evaluating the role and optimal systemic therapy after complete resection of the primary bone tumor. In the absence of definitive treatment guidelines, therapy should be chosen based on the patient’s age, overall health, functional status, and the suspected biological causes of the disease.

In healthy bone, cross-talk between osteoblasts and osteoclasts is a tightly regulated process to maintain a healthy bone matrix.^14^ At a systemic level, regulation is influenced by parathyroid hormone, vitamin D, PTH-related peptide, calcitonin, and corticosteroids. Within the bone, osteoclast-derived RANK ligand (L) inhibits bone formation by osteoblasts, and its effect is tightly regulated by secretion of osteoprotegerin, which functions as an inactivating decoy receptor.^15^

In GCTB, sheets of mononuclear cells of myeloid lineage are mixed with occasional multinucleated giant cells. These giant cells express RANK and have been repeatedly reported to be of osteoclastic origin. Several reports have suggested that their development is reactive in nature^16^ and that the true neoplastic compartment is of mesenchymal origin, with minimal differentiation along the osteoblastic lineage.^15^ These neoplastic mesenchymal cells secrete RANK ligand and are thought to be the source of activation and continued stimulation of RANK-expressing osteoclasts and giant cells,^17^ thus conferring the bone lytic phenotype. These osteoblastic precursor cells further enhance their destructive potential by recruitment of immune cells and increased vascularization. However, the reversal of the histological bone lytic phenotype with anti-RANK-L monoclonal antibody therapy implicates RANK-L as a key factor in the formation of these tumors. It is, thus, hypothesized that inhibition of RANK-expressing osteoclasts by the fully human antibody denosumab would ameliorate the osteolytic phenotype. By reducing bioavailable RANK ligand through binding and elimination by the immune system, osteoclastic activity was thought to be inhibited, and this intervention in turn could balance the impaired equilibrium between bone formation and destruction. Secondary effects of denosumab, including decrease in vascularization and recruitment of secondary immune cells, would appear plausible but are still subject to further investigation.^18^ The safety and efficacy of denosumab has been tested in proof-of-concept phase II clinical trials,^1^ demonstrating radiographic and histological metastatic tumor responses in patients with GCTB.

The underlying genetic or molecular events leading to enhanced RANK ligand secretion by GCTBs are yet to be fully explained. The molecular changes enabling a subgroup of these tumors to spread metastatically are equally unclear. By RECIST criteria, the patient in the current case report achieved a partial radiographic response. More intriguingly, histological transformation into a benign fibroblastic phenotype with disappearance of giant cells was observed on lung core biopsy after treatment with denosumab. After the initial size reduction, the metastatic lung lesions have remained stable for more than a year since initiation of systemic therapy with denosumab. It is unclear whether the giant cell tumor phenotype is reversed or merely suppressed by RANK ligand inhibition. Understanding this would have significant clinical implications by guiding the optimal length of therapy. It is not known if therapy for metastatic GCTB should be guided by imaging studies alone or in combination with histological analysis of metastatic lesions after therapy with denosumab. The safety and tolerance profile of denosumab for metastatic GCTBs mirrors the acceptable safety profile in other approved indications such as treatment of osteoporosis and prevention of skeletal-related events in patients with solid tumors with skeletal metastases. Therefore, it seems sensible to continue maintenance systemic therapy with denosumab in patients with metastatic GCTB in the absence of unmanageable side effects or progression of metastatic disease. Further data from prospective randomized clinical trials are needed to help the clinician choose the optimal treatment for metastatic GCTBs.
